# Weight Loss Trajectories After Bariatric Surgery for Obesity: Mathematical Model and Proof-of-Concept Study

**DOI:** 10.2196/13672

**Published:** 2020-03-09

**Authors:** Chloe Dimeglio, Guillaume Becouarn, Philippe Topart, Rodolphe Bodin, Jean Christophe Buisson, Patrick Ritz

**Affiliations:** 1 Centre Hospitalier Universitaire de Toulouse Toulouse France; 2 Clinique de l'Anjou Angers France; 3 Hôpital d'Instruction des Armées Robert Picqué Service de Santé des Armées Bordeaux France; 4 École Nationale Supérieure d'Électrotechnique, d'Électronique, d'Informatique, d'Hydraulique et des Télécommunications Toulouse France

**Keywords:** weight changes, obesity or bariatric surgery, weight regain, modeling trajectories

## Abstract

**Background:**

Obesity surgery has proven its effectiveness in weight loss. However, after a loss phase of about 12 to 18 months, between 20% and 40% of patients regain weight. Prediction of weight evolution is therefore useful for early detection of weight regain.

**Objective:**

This proof-of-concept study aimed to analyze the postoperative weight trajectories and to identify “curve families” for early prediction of weight regain.

**Methods:**

This was a monocentric retrospective study with calculation of the weight trajectory of patients having undergone gastric bypass surgery. Data on 795 patients after a 2-year follow-up allowed modeling of weight trajectories according to a hierarchical cluster analysis (HCA) tending to minimize the intragroup distance according to Ward. Clinical judgement was used to finalize the identification of clinically relevant representative trajectories. This modeling was validated on a group of 381 patients for whom the observed weight at 18 months was compared to the predicted weight.

**Results:**

Two successive HCA produced 14 representative trajectories, distributed among 4 clinically relevant families: Of the 14 weight trajectories, 6 decreased systematically over time or decreased and then stagnated; 4 decreased, increased, and then decreased again; 2 decreased and then increased; and 2 stagnated at first and then began to decrease. A comparison of observed weight and that estimated by modeling made it possible to correctly classify 98% of persons with excess weight loss (EWL) >50% and more than 58% of persons with EWL between 25% and 50%. In the category of persons with EWL >50%, weight data over the first 6 months were adequate to correctly predict the observed result.

**Conclusions:**

This modeling allowed correct classification of persons with EWL >50% and could identify early after surgery the patients with potentially less that optimal weight loss. Further studies are needed to validate this model in other populations, with other types of surgery, and with other medical-surgical teams.

## Introduction

Obesity surgery has proved to be effective in the long term for weight loss and for remission or improvement of comorbidities associated with excess weight. In the long term, however, weight regain occurs in 20% to 40% of patients [1-7], and this is a major factor in the recurrence of comorbidities.

Many factors have been studied to predict weight regain but few have been found to be relevant [8]. Ritz et al [9] showed that patients in failure at 2 years according to the Reinhold criteria [10] developed an unfavorable weight trajectory from the early postoperative period. Careful monitoring of early postoperative weight trajectories could thus be one way to achieve early identification of patients with weight regain. Ideally, self-monitoring of weight could empower patients to alert health care providers sufficiently early so that solutions can be studied.

Wise et al [11] have shown that an exponential decay can describe the weight path in the 3 months after gastric bypass. They used excess weight loss (EWL) and did not show a long-term 5-year prediction value of the classification. EWL calculations can be difficult for patients who are not used to this arithmetic. An easy-to-use application for computing weight paths using only weight would help patients in monitoring their weight loss.

This proof-of-concept study aimed to analyze the postoperative weight trajectories and identify “curve families” for early prediction of weight regain. We combined a method of classification of unsupervised curves and clinical expertise to define the reference weight trajectories after gastric bypass surgery.

## Methods

### Study Design

This was a monocentric retrospective study modeling the weight trajectories of patients having undergone gastric bypass surgery.

### Ethics

All procedures performed in this study involving human participants were in accordance with the ethical standards of the institutional and/or national research committee and the 1964 Declaration of Helsinki and its later amendments or comparable ethical standards.

### Patients

Group 1 comprised 795 patients who had gastric bypass surgery between 2003 and 2012 with at least 2 years of follow-up in the same reference center, performed by two surgeons (GB and PT). They were included for modeling their weight trajectories. They were within the Haute Autorité de Santé criteria for obesity surgery [12]. The patients analyzed represented 63.00% (795/1262) of the initial patients. A total of 11.01% (139/1262) were excluded for redo surgery; 28.62% (320/1118) were excluded because they were lost to follow-up within the 2 years after surgery.

Group 2 comprised 381 patients with a follow-up of 5 years after gastric bypass. They were classified according to the Reinhold criteria and allowed us to assess predictive capability of the model when applied within the first 18 months to predict later weight.

### Evaluation Criteria for Weight Loss

Weight was measured with patients wearing undergarments during postsurgery follow-up visits (1, 3, 6, 9, 12, and 18 months). The reference weight was that of the day before surgery. Certain data were missing. The EWL was calculated based on a body mass index (BMI) of 25 kg/m^2^, with equation EWL = (initial weight – actual weight)/(initial weight – 25* height^2^); weight in kg and height in meters. The Reinhold [10] criteria were used to define failure (EWL after 18 months of less than 25%), success (EWL after 18 months exceeding 50%), and an intermediate result (EWL between 25% and 50%).

### Calculations and Statistics

Calculations were performed using R version 3.0.2 (R Foundation for Statistical Computing) and MATLAB version 2010a (The MathWorks, Inc).

#### Objectives

The main objective was to establish weight trajectories representative of all trajectories in group 1 (795 patients). After imputation for missing values, all the initial trajectories were clustered into an optimal number *y* of trajectory groups. One representative trajectory was extracted for each group of trajectories. All the representative trajectories were merged into a smaller number of families according to the behavior of the trajectories evaluated by clinical expertise. The validity of this collection of representative trajectories was evaluated using the weight trajectories of group 2.

#### Management of Missing Data

The proportion of missing data was 9.4% at 1 month, 56.7% at 3 months, 51.7% at 6 months, 37.1% at 9 months, 35.6% at 12 months, and 38.7% at 18 months. The missing data were replaced by multiple imputation [13] in 5 iterations based on age, sex, presurgery weight, height, and the presence of diabetes.

#### Creation of a Catalog of Weight Trajectories Representative of the Base

To constitute trajectory groups that maximize the interclass distance and minimize the intraclass distance (the Ward method), successive hierarchical cluster analyses (HCA) without a priori on the number of classes that were used [14]. The number of classes retained (*x*) after the first HCA was that of preceding an overly heterogeneous association (while optimizing distance of Ward). Starting from this first classification, one representative curve per class was extracted using a criterion of representativity of the group, according to Dimeglio [15]. We thus obtain *x* representatives.

We performed as many additional HCA as necessary on this base of *x* representatives to continue reducing the number of classes to a clinically acceptable level, which we defined here as a maximum of 15 classes. Here *y* is the number of representative trajectories obtained at the last iteration. These *y* representatives constituted the reference base of weight trajectories.

#### Clinical Classification of Representative Trajectories

The final *y* representative curves were grouped into a number of families according to the behavior of weight trajectories evaluated by clinical expertise (gradual decrease, weight rebound, etc).

#### Validation of Representative Trajectories

**Figure 1:**
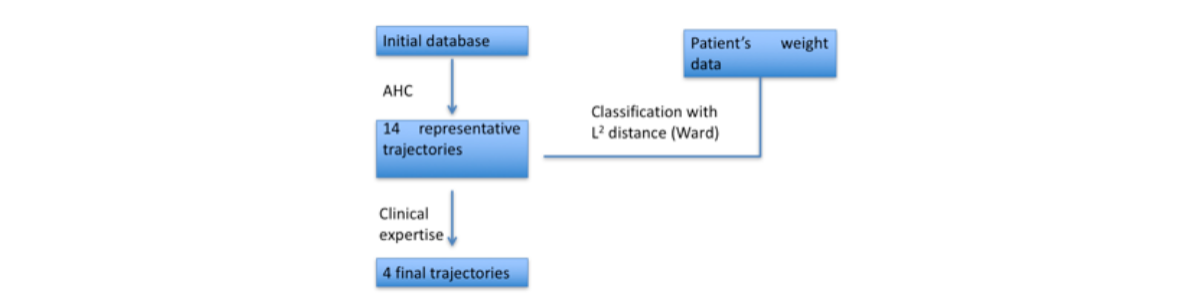
Graphic illustration of the procedure.

For a given patient, their weight trajectory was brought closer to one of the *y* representative trajectories by minimizing the distance between them [16]. Figure 1 gives a graphic illustration of the procedure.

#### Application to the Weight Trajectories of Group 2

**Figure 2:**
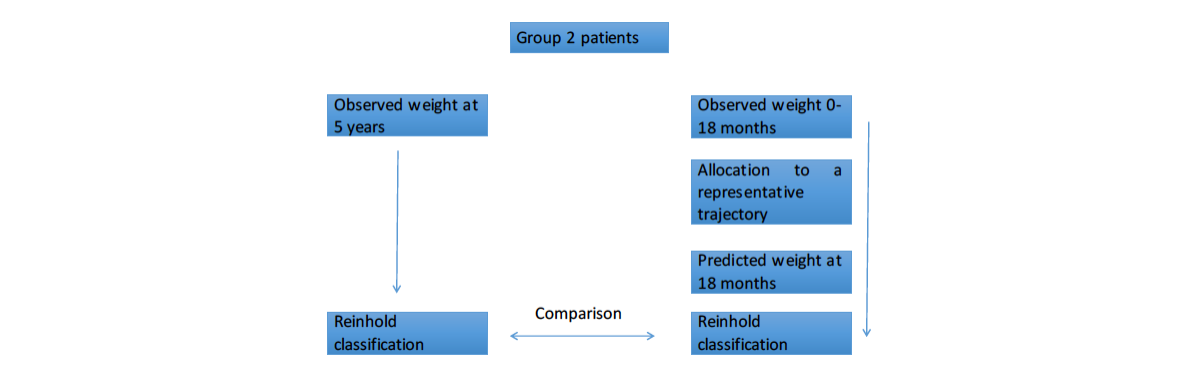
Procedure for validation of trajectories in patients in group 2.

To determine the predictive validity of the reference base (the *y* representative trajectories), the procedure was applied to the 381 patients in group 2. Each patient was associated to a representative trajectory that predicted EWL at 18 months according to the Reinhold classification [10]. Similarly, the Reinhold classification was applied to the weight result observed at 5 years. The two values obtained after these Reinhold classifications were compared. Figure 2 illustrates the procedure. To estimate the variability of our classification results, we chose to randomly extract 100 times 100 trajectories among the 381 patients in group 2 (bootstrap procedure). Thus, we compared 100 times the known identification of 100 patients to that obtained by bringing them closer to one of the representative trajectories.

## Results

### Constitution of Weight Trajectories Representatives

**Figure 3:**
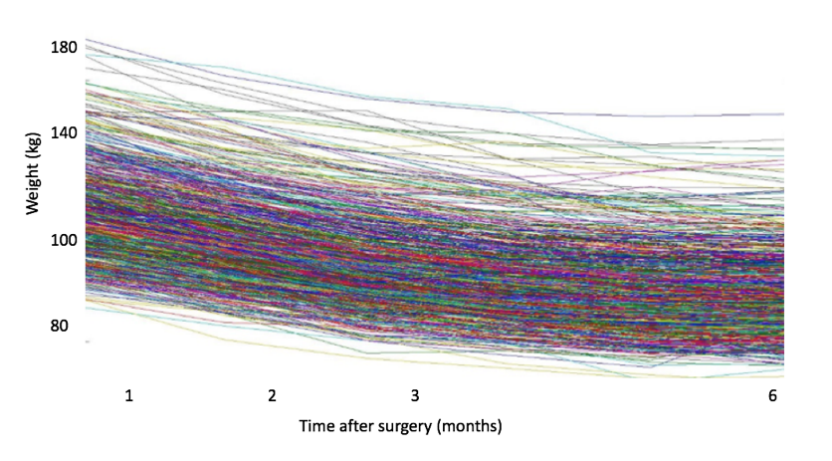
Weight trajectories after surgery in group 1 (n=795).

**Figure 4:**
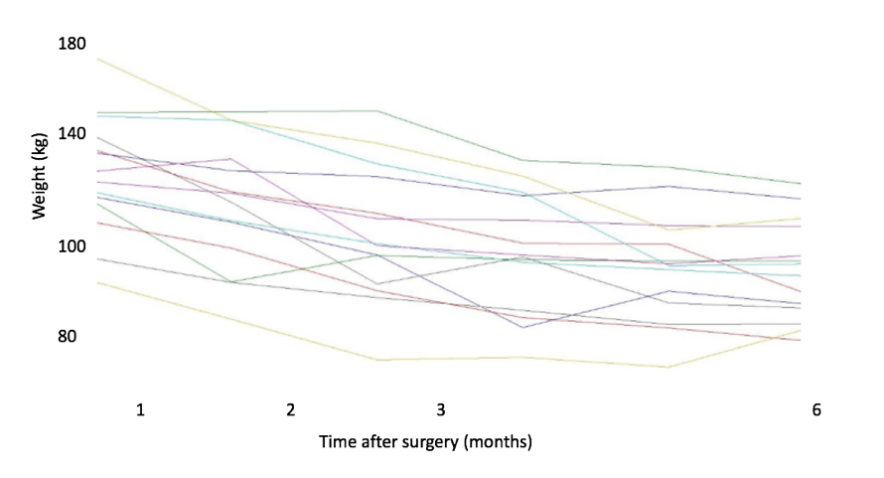
Weight trajectories after two hierarchical cluster analyses in group 1 (n=795).

A total of 795 patients were included in this study (141 men and 654 women). The variability of data did not allow us to summarize the information in a single representative trajectory (Figure 3). Homogeneous groups of trajectories were necessary to summarize the entirety of the information. A total of 56 classes of representative trajectories came from the first HCA. After two successive HCA, the number of representative trajectories was reduced to 14 classes. Figure 4 shows the trajectories of the representative curve of each class.

### Clinical Classification of Representative Trajectories

**Figure 5:**
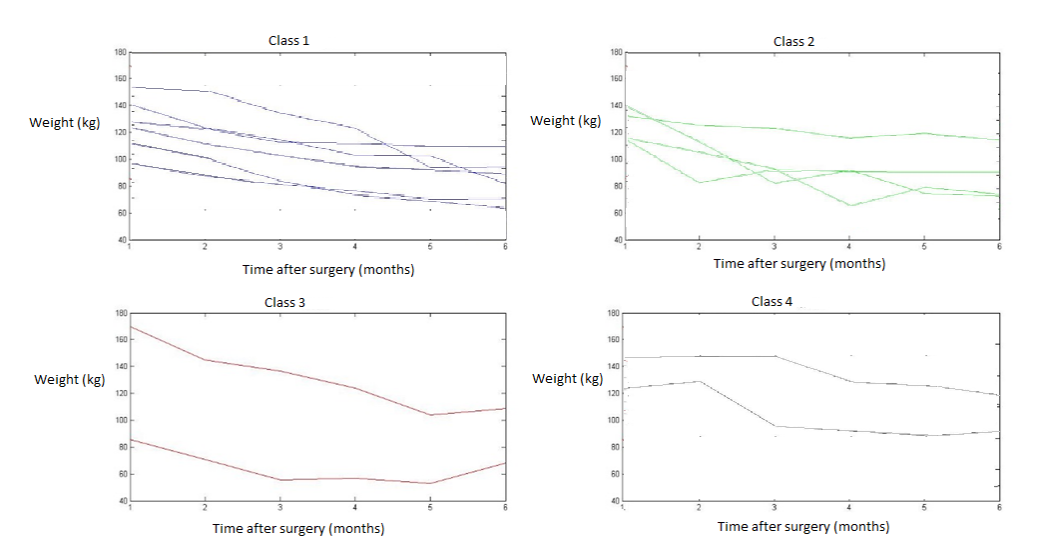
The four families of weight trajectories.

Several of these representative trajectories had the same behavior (Figure 5):

- Weight decreased systematically over time or decreased and then remained stable (class 1). This family includes 6 of the 14 trajectories.
- Weight decreased, then increased, and then decreased again (class 2). This family includes 4 of the trajectories.
- Weight decreased and then increased (class 3). This family includes 2 of the 14 trajectories.
- Weight stagnated at first and then started to decrease (class 4). This family includes 2 of the 14 trajectories.

Preoperative factors (age, sex, presence of diabetes) did not modify the trajectory predictions.

### Application to the Weight Trajectories of Group 2

The Reinhold classification applied to weight observed at 18 months identified 87.4% (333/381) of patients in the category EWL >50% and 12.6% (78/411) of patients in the category EWL between 25% and 50%. There were no patients in the category EWL <25%.

The Reinhold classification was applied to the estimated weight at 18 months based on representative trajectories. Only one trajectory (7.1%) was classified EWL <25%. Six trajectories (42.9%) were classified EWL between 25% and 50%. Seven trajectories (50%) were classified EWL >50%.

The match between the Reinhold classification [10] based on observed or predicted weight is shown in Table 1. Classification with reference trajectories produced an overall rate of correct classification of more than 93%. It allowed correct classification of 97.6% (325/333) of persons with EWL >50% and more than 59% (46/78) of persons with EWL between 25% and 50%.

Table 2 shows the influence of the number of early weight values used in the prediction on the results at 5 years. The more the number of points increased, the more precise was the classification of the different profiles (best mean and lowest variability). For the persons who would be in the category EWL >50% at 18 months, the match rates increased with the number of values over time, but as of 6 months postsurgery, 91.6% (305/333) were well classified.

For the persons who would be in the category EWL between 25% and 50%, the match rate showed little increase over time (going from 47% to 56%). Therefore, between 44% and 53% of patients were poorly classified by the trajectories.

**Table 1:** Match between the Reinhold classification applied to observed and predicted weight of persons in group 2.

EWL calculated with observed weight		Predicted weight, mean (SD)		
		EWL^a^ >50%	EWL 25%-50%	EWL <25%
Observed weight, mean (SD)	EWL >50%	97.6 (1.49)	2.37 (1.49)	0
Observed weight, mean (SD)	EWL 25%-50%	41.3 (12.8)	58.7 (12.8)	0

^a^EWL: excess weight loss.

**Table 2:** Influence of the number of early weight values used in the prediction of weight identification at 5 years. The mean numbers in the columns are the percentages of patients correctly classified.

EWL calculated with observed weight		Predicted weight with 6 months data mean (SD)			Predicted weight with 9 months data mean (SD)			Predicted weight with 12 months data mean (SD)	
		EWL^a^ >50%	EWL 25%-50%	EWL >50%		EWL 25%-50%	EWL >50%		EWL 25%-50%
Observed weight, mean (SD)	EWL >50%	91.6 (2.96)	8.4 (2.96)	96 (1.86)		4 (1.86)	97.4 (1.6)		2.6 (1.6)
Observed weight, mean (SD)	EWL 25%-50%	52.7 (13.3)	47.3 (13.3)	46.3 (13.9)		53.7 (13.9)	43.7 (13.5)		56.3 (13.5)

^a^EWL: excess weight loss.

## Discussion

### Principal Findings

This study shows that it is possible to model weight loss over the 18-month period after a gastric bypass in representative trajectories. Applied to a group of patients at 5 years postsurgery, the representative trajectories correctly predicted more than 93% of the values. Postoperative data available for 1 to 6 months are sufficient to provide a satisfactory prediction of EWL >50%.

### Strengths and Limitations

Unsupervised classification methods like the HCA that we used here makes it possible to define families of trajectories without a priori on the number of classes to obtain. This is a very commonly used method. In this study, it allowed us to determine families of weight trajectories only from their general appearance and without using additional variables, like in the research of Wise [11]. One of the advantages is that the classification can be updated as new weight data are added to the previous one. Also, we chose to use weight and not EWL (or the percentage of weight loss) to develop the trajectories because they are accompanied by arithmetic biases. For example, for a very high initial BMI, EWL will be less than for a lower BMI, despite an equivalent weight loss. We only used EWL to compare the value predicted by the trajectory to the observed value in group 2. The concept of predicting later weight is therefore valid from this data. The use of weight trajectories rather than trajectories of EWL or of percentages of weight loss was also preferred for conserving the maximum of behaviors within the trajectories.

The cohort came from the same center with two surgeons and a formalized protocol for the preparation and follow-up of patients. However, that limits the variability introduced by multiple teams using different preparation and follow-up protocols. This study did not analyze known factors of failure or of inadequate weight result like those identified by other authors [11]. The prediction is better for the patients who lose a lot of weight. This is explained by the initial imbalance between the three classes based on EWL in the database. Further analyses on more balanced bases are needed.

An early prediction of weight trajectory is clinically useful. A success prediction may empower postsurgical patients to carry on and reassure them, as many are anxious about regaining weight because of their past history of weight loss failures. This does not mean that weight will no longer be monitored. This also suggests that if a person is not classified as a later success, every effort should be made to understand the reasons and implement early corrective strategies. In that sense, the factors classically associated with less than optimal weight loss can be used not as predictors (with little efficiency) but as targets for an early additive strategy. Despite the low power to predict future results in this category, the idea of an early alert is clinically useful. Validation studies on other cohorts are needed. We should also point out that the validation database was very imbalanced (87.4% of EWL >50% vs 12.6% with EWL between 25% and 50% and no patient in weight loss failure). It is possible that the classification of patients with average weight loss will improve with better-balanced validation database. A machine learning process added to the application would provide evolutive classifications as the amount of data increases [17-19].

### Analysis of the Results

This study shows that a hierarchical cluster analysis allowed us to identify 4 profiles of weight trajectories associated with clinical expertise. The patients who were the most successful were those who lost weight regularly. The persons who lost the least had difficulties in the initial phase or had a secondary weight regain.

None of the preoperative parameters differed between the families of curves. This agrees with other published studies [8]. It seems difficult to predict weight loss after surgery with factors that do not consider the capacity for personal, psychological, or metabolic adaptation during the postsurgery period. The factors classically associated with poor weight loss results are postoperative (recurrence of depression, eating disorders, lack of follow-up, lack of arrangements for changes of eating habits, and physical activity) and usually occur late after surgery (24 months). The early identification of a trajectory is therefore an interesting feature of this model. Applied to a population of persons having undergone surgery and at 18 months after this surgery, this classification correctly identified more than 97% of EWL >50% and more than 58% of EWL between 25% and 50%.

Wise et al [11] recently published a comparable study. In a retrospective analysis of a cohort of gastric bypassed patients followed for 3 years, the authors demonstrated the exponential nature of the decrease of EWL and analyzed the factors influencing the trajectory, such as age. Our study used an original mathematical approach and adds to the study of Wise et al [11] because it validates the concept of trajectories in an independent population at 5 years. Like Wise et al [11], we conducted the analysis using EWL without showing better results than with weight only.

### Clinical Relevance

Given the prevalence of weight regain after obesity surgery, early identification of a nonoptimal weight trajectory would help to strengthen the second lines of treatment by reinforcing measures for behavioral change (self-care, physical activity, nutrition). This study proves the concept that a simple application could allow surgical patients to enter weight data themselves, evaluate the result, and eventually alert the health care provider.

## Abbreviations

BMI: body mass index
EWL: excess weight loss
HCA: hierarchical cluster analysis
